# Potential Roles of *Dec* and *Bmal1* Genes in Interconnecting Circadian Clock and Energy Metabolism

**DOI:** 10.3390/ijms19030781

**Published:** 2018-03-08

**Authors:** Fuyuki Sato, Akira Kohsaka, Ujjal K. Bhawal, Yasuteru Muragaki

**Affiliations:** 1 Department of Pathology, Wakayama Medical University School of Medicine, 811-1 Kimiidera, Wakayama 641-8509, Japan; ymuragak@wakayama-med.ac.jp; 2 Department of Physiology, Wakayama Medical University School of Medicine, 811-1 Kimiidera, Wakayama 641-8509, Japan; kohsaka@wakayama-med.ac.jp; 3 Department of Biochemistry and Molecular Biology, Nihon University School of Dentistry at Matsudo, 2-870-1 Sakae-cho Nishi, Matsudo, Chiba 271-8587, Japan; bhawal.ujjal.kumar@nihon-u.ac.jp

**Keywords:** *Dec1*, *Dec2*, *Bmal1*, clock gene, energy metabolism

## Abstract

The daily rhythm of mammalian energy metabolism is subject to the circadian clock system, which is made up of the molecular clock machinery residing in nearly all cells throughout the body. The clock genes have been revealed not only to form the molecular clock but also to function as a mediator that regulates both circadian and metabolic functions. While the circadian signals generated by clock genes produce metabolic rhythms, clock gene function is tightly coupled to fundamental metabolic processes such as glucose and lipid metabolism. Therefore, defects in the clock genes not only result in the dysregulation of physiological rhythms but also induce metabolic disorders including diabetes and obesity. Among the clock genes, *Dec1* (*Bhlhe40*/*Stra13*/*Sharp2*), *Dec2* (*Bhlhe41*/*Sharp1*), and *Bmal1* (*Mop3*/*Arntl*) have been shown to be particularly relevant to the regulation of energy metabolism at the cellular, tissue, and organismal levels. This paper reviews our current knowledge of the roles of *Dec1*, *Dec2*, and *Bmal1* in coordinating the circadian and metabolic pathways.

## 1. Introduction

The supply and demand of cellular bioenergy vary across the 24 h light-dark cycle in mammals. This phenomenon is subject to the circadian clock system [1,2,3,4,5,6], which comprises the central clock in the suprachiasmatic nucleus (SCN) of the hypothalamus and peripheral clocks that reside in extra-SCN areas of the brain and in peripheral tissues. Both the central and peripheral clocks are made up of similar clock machinery, the so-called molecular clock, which consists of a series of clock genes [7,8,9,10]. Since their discovery as components of a circadian oscillator, the clock genes have been shown to regulate cellular energy metabolism and body energy homeostasis through the transcriptional control of metabolic genes, whose function has a great influence on cellular redox and bioenergy [1,3,4,5,6]. Of interest, it has also been shown that the function of clock genes is in turn altered by changes in energy balance [2,4,11,12], suggesting the mutual regulation of the circadian and metabolic systems. Here, we offer a description of the interplay between the circadian and metabolic systems, focusing especially on the key clock genes *Dec1*, *Dec2*, and *Bmal1*, which have been shown to be linked to energy metabolism, and insights into their emerging roles in the understanding of circadian and metabolic disorders.

## 2. The Circadian Clock Machinery

**Figure 1:**
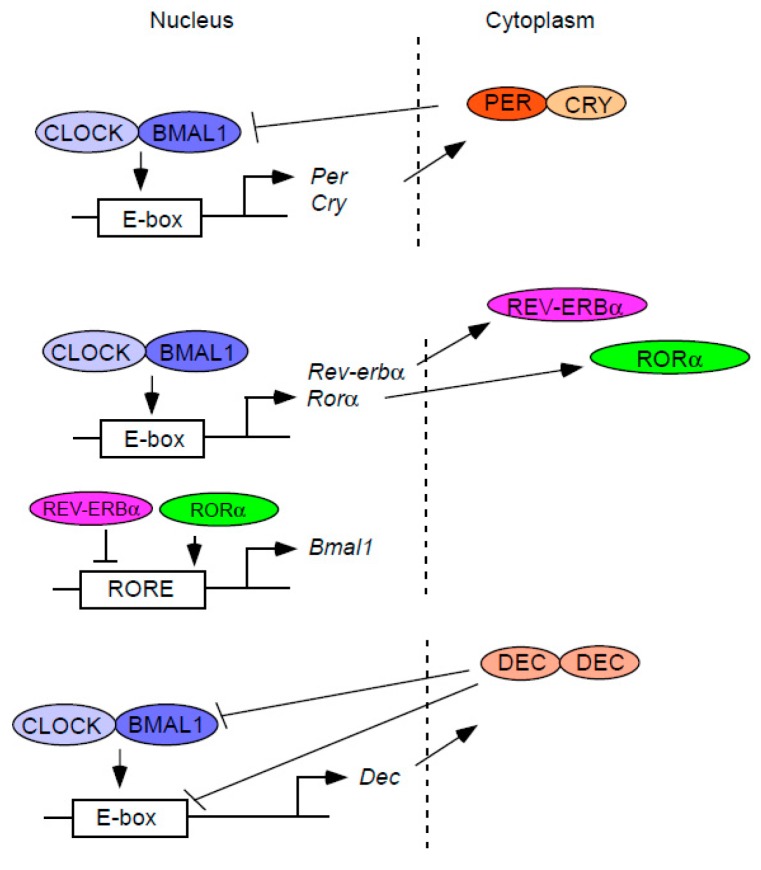
The core molecular mechanism of the circadian clock. The molecular clock is composed of diverse autoregulatory feedback loops. The core components of the molecular clock, CLOCK and BMAL1, activate the transcription of repressor clock genes such as *Per*, *Cry*, *Rev-erbα*, and *Dec*. CLOCK and BMAL1 also activate the transcription of inducer *Rorα*. The protein products of these repressor or inducer genes inhibit or promote their own transcription in distinct ways, thus forming various feedback loops that create a coordinated circadian rhythm at the gene expression level.

Circadian clock genes are defined as genes that are required to regulate circadian rhythms of physiology at the cellular, tissue, and organismal levels. Clock genes form the molecular clock which is composed of interconnected feedback loops of gene transcription and translation (Figure 1). In mammals, two core clock genes, *Clock* and *Bmal1*, encode proteins that are members of the basic helix-loop-helix (bHLH)-PAS transcription factor family [13,14,15,16]. Through protein-protein interactions involving their PAS domains, CLOCK and BMAL1 form a heterodimeric complex in the cytoplasm, which then translocates to the nucleus and binds to E-box enhancer sequences to activate the transcription of repressor clock genes [13,17]. These include the Period (*Per1* and *Per2*) and *Cryptochrome* genes (*Cry1* and *Cry2*), whose protein products lower their own transcription by repressing CLOCK:BMAL, thus composing an autoregulatory feedback loop. Since the discovery of this “core” feedback loop, additional feedback loops have also been described. By binding to E-box enhancers, CLOCK:BMAL1 activates the transcription of another core clock gene, *Rev-erbα*, which codes for an orphan nuclear receptor [18,19]. The REV-ERBα protein in turn inhibits *Bmal1* transcription by direct binding to the ROREs (retinoic acid-related orphan receptor response elements) residing in the *Bmal1* gene promoter, thereby forming another feedback loop [18,19].

The *Dec1* and *Dec2* genes, which encode bHLH transcription factors, have also been proposed as clock genes that comprise an additional autoregulatory feedback loop [20,21,22,23]. *Dec1* and *Dec2*, similar to the *Rev-erbα* gene, are transactivated by CLOCK:BMAL1 through E-box elements in their promoters [21,22,23]. However, unlike the REV-ERBα protein, which inhibits its own expression by repressing the transcription of one of its activators, *Bmal1*, DECs repress their own transcription by directly binding to the BMAL1 protein and/or by competing with CLOCK:BMAL1 for the occupancy of E-box sequences in their promoters [20,21,22,23,24,25]. As repressors of CLOCK:BMAL1-induced transactivation, DECs also inhibit the transcription of other clock and clock-controlled genes, such as *Per1* and *Dbp* (albumin d-element binding protein) [20,22,26]. Collectively, the molecular clock is composed of a complex network of autoregulatory feedback loops of gene transcription and translation, thus generating circadian rhythms at the molecular level.

## 3. The Diurnal Rhythm of Energy Metabolism and Its Significance

The function of a wide range of metabolic processes, such as the synthesis and breakdown of carbohydrates, lipids, and proteins varies across the sleep/wake cycle in mammals. For instance, glycogen synthesis in the muscle and liver occurs during the waking period, whereas glycogenolysis in the liver peaks during sleep [27,28]. An increase in lipoprotein lipase activity, which promotes lipid absorption from the blood-stream into tissues, also occurs during the fed (i.e., waking) state [29]. One benefit of these metabolic rhythms is that it enables metabolic tissues to anticipate supply and demand of energy in the body and optimize both energy storage and utilization. Therefore, it is conceivable that uncoupling the energy supply from its demand in each metabolic organ results in a malfunction of body energy metabolism in mammals. Indeed, both clinical and experimental studies have long suggested that disruption of behavioural rhythms such as sleep/wake and fasting/feeding cycles leads to defects in glucose and lipid metabolism. Best known are the epidemiological findings that sleep disturbances and shift work are highly associated with the development of obesity, dyslipidaemia, and type 2 diabetes in humans [30,31,32,33,34,35,36,37,38]. Similarly, in rodents, it has also been reported that a shift in meal time not only disturbs the behavioural rhythm but also induces insulin resistance [39,40]. *Dec1* knockout mice showed behavioral rhythm with longer circadian period and affected the circadian phase of *Per1* [26]. In addition, double mutant mice of *Per1* and *Dec* revealed synergistic effects of circadian disturbance [41]. We demonstrated that DECs have the strongest suppressive effects on CLOCK:BMAL1-induced transactivation among negative regulators DECs, PERs and CRYs [20,21,22]. Recent our reports using *Dec* knockout mice showed regarding the role of Dec in metabolism. First, *Dec1* knockout mice decreased lipid levels and oxidative stresses, increasing fibroblast growth factor 21 (FGF21) levels [42]. Second, we revealed dominant energy sensor phosphorylation AMP-activated protein kinase (pAMPK), which mention at later Section 6, is markedly increased in *Dec2* knockout mice livers [43]. These evidences suggest that *Dec* play important roles in the regulation of circadian rhythm and metabolism.

It has been proven that Clock mutant mice disturbed diurnal feeding rhythm and developed a metabolic syndrome [44]. It has also been reported that DEC2 amino-acid mutation P385R is associated with short sleep [45]. On the other hand, *Dec2* knockout mice did not reveal the sleep phenotype [46]. It seems that DEC2 P385R works as a dominant negative fashion. We also revealed that human DEC1 amino-acid mutation R65A worked as a dominant negative fashion against CLOCK:BMAL1 transactivation [21]. These dominant negative functions of DEC mutation may affect differences in sleep phenotype of *Dec* knockout mice. Collectively, these human and animal studies strongly suggest a significant role of metabolic and circadian rhythms in maintaining health and preventing metabolic and circadian disorders.

## 4. Clock Genes Regulate Both Metabolic Rhythms and Processes

As the molecular mechanism of the mammalian circadian clock was revealed, it became clear that metabolic rhythms are under the control of the molecular clock [1,2,3,4,5,6]. For example, in studies of with tissue-specific knockout of the clock gene, the diurnal rhythm in hepatic glucose metabolism was found to be regulated by the liver clock [44,47]. Recent studies have also shown that the production of hepatic metabolites such as nucleotides and amino acids exhibits diurnal variation in mice and that BMAL1 plays an important role in the circadian oscillation of these metabolites [48]. In addition to the liver clock, the molecular clock in the pancreas was found to regulate the daily rhythm of insulin secretion [49]. While these local clocks play an important role in generating local metabolic rhythms, systemic signals produced by the fasting/feeding cycle, which is driven by the SCN clock, also affect certain cellular metabolic rhythms independently of the local clock [50].

Apart from its role in generating metabolic rhythms, the molecular clock also participates in fundamental metabolic processes such as the proliferation of islet cells in the pancreas, glucose transport by skeletal muscle, and adipogenesis in white adipose tissue. Both the BMAL1 and CLOCK proteins in the pancreas have been shown to regulate secretion of insulin and proliferation of pancreatic islets [51,52]. Insulin-dependent glucose uptake in skeletal muscle was also found to be associated with the function of the *Bmal1* gene [53]. Furthermore, BMAL1 regulates adipocyte differentiation and lipogenesis in white adipose tissue and plays an important role in ectopic fat deposition in the liver and skeletal muscle [54,55]. Intriguingly, both genetic and epigenetic in silico studies support these experimental findings that the *Bmal1* gene is linked to energy metabolism. Human genome-wide association studies have revealed that certain haplotypes of the *Bmal1* gene are associated with the development of type 2 diabetes [56]. Moreover, using whole-genome chromatin immunoprecipitation-based analyses, a large number of BMAL1 target genes were found to be classified as genes that are related to cellular energy metabolism [57,58,59].

Given that BMAL1, together with CLOCK, directly activates the transcription of both *Dec1* and *Dec2* [20,21,22,23], it is possible that BMAL1 may control energy metabolism through the function of DEC1 and DEC2. Although this possibility remains to be examined, the DECs per se regulate lipid metabolism in metabolic tissues. DEC1 and DEC2 inhibit lipogenesis in the liver by repressing the transcription of *Srebp-1c* (sterol regulatory element-binding protein-1c), which codes for the master regulator of lipogenic genes, by binding to the E-box in its promoter region [60,61]. Therefore, changes in the DEC levels could affect the degree of lipid storage in the liver. Indeed, both leptin-deficient *ob*/*ob* and leptin receptor-deficient *db*/*db* mice show a decrease in *Dec1* expression in the liver and a significant increase in hepatic triglycerides [61], whereas only a mild increase in hepatic triglyceride content was observed in mice with alcohol-induced fatty liver, in which *Dec1* expression was increased [62]. White adipose tissue also requires the function of the *Dec1* gene to regulate adipogenesis. DEC1 suppresses adipocyte differentiation by inhibiting the expression of peroxisome proliferator-activated receptor γ (*Pparγ*), a gene encoding a nuclear receptor critical for adipogenesis [63]. Collectively, these findings suggest that, in addition to its role as a circadian oscillator, the molecular clock plays an important role in regulating fundamental properties of energy metabolism in major metabolic organs.

## 5. The Clock Senses Energy Balance

While the molecular clock regulates energy metabolism, changes in energy balance, in turn, affect the function of the molecular clock [64]. For example, mice fed a high-fat diet exhibit an increase in the circadian period of locomotor activity under constant dark conditions, which indicates that high-fat feeding alters the function of the SCN clock [65]. Energy excess also attenuates the amplitude of rhythmic expression of clock genes in peripheral tissues [12,65]. In addition to excess energy intake, the timing of energy intake has been shown to impact the rhythmic expression of clock genes in metabolic tissues [66]. Specifically, in mice, when food availability is restricted only to the light (i.e., sleep) phase, the rhythmic expression of clock genes occurs in anti-phase to that observed in ad lib feeding. Although the molecular mechanism is not fully understood, this phenomenon is predominantly observed in peripheral metabolic tissues, such as the liver and white adipose tissues, and not in the SCN [39,67]. It should be noted that changes in the fasting/feeding cycle not only affect patterns of rhythmic expression of clock genes but also alter their expression levels. In the murine liver, the mRNA expression level of *Dec1* is significantly decreased by fasting and increased by re-feeding [68,69,70], although *Dec2* expression is not altered by feeding [68,69]. The expression level of *Per2* in the liver, but not in the heart and lung, is also increased by re-feeding after fasting [68,70]. Although the effects of changes in the fasting/feeding cycle on clock gene expression are limited to certain clock genes and to specific tissues, these studies nevertheless suggest that the molecular clock is sensitive to alteration in the balance and the timing of energy intake.

## 6. Molecules at the Intersection of the Molecular Clock and Energy Metabolism

How does the molecular clock sense changes in energy balance? The fasting/feeding and activate/resting cycles induce a dynamic change in the levels of metabolites and fuels in the cells and interstitial spaces surrounding the cells. The molecules with altered levels include cellular redox components and energy resources, energy substrates and oxygen. Growing evidence now suggests the possibility that any of these molecules can be sensed by the molecular clock through molecules that not only function as key metabolic regulators but also modulate clock function.

### 6.1. Cellular Redox

**Figure 2:**
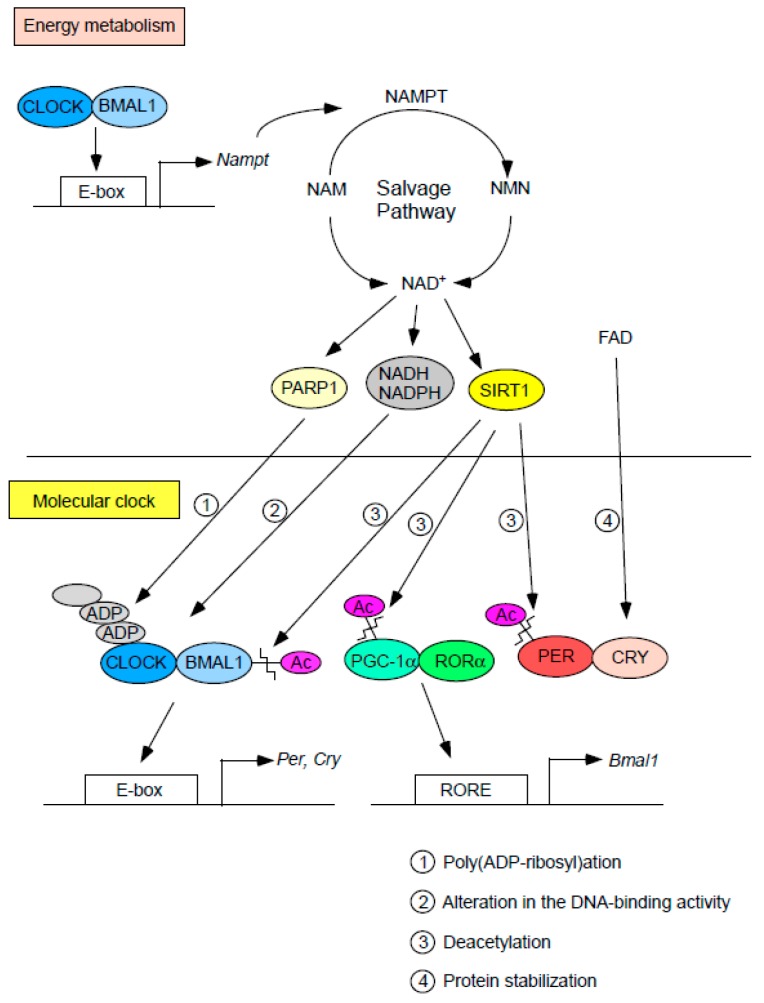
Interplay between cellular redox factors and the molecular clock. Cellular redox status directly and indirectly affects the function of the molecular clock through changes in the levels of cofactors (i.e., NADH, NADPH, and FAD) and/or the activity of NAD^+^-consuming enzymes (SIRT1 and PARP1). The molecular clock in turn regulates the transcription of the *Nampt* gene, which encodes the rate-limiting enzyme in the NAD^+^ salvage pathway.

Molecules associated with the cellular redox state were perhaps the first metabolic factors to be proposed to influence the function of the molecular clock (Figure 2). Specifically, cellular levels of the hydrogen carriers NAD^+^ (nicotinamide adenine dinucleotide) and FAD (flavin adenine dinucleotide) have been shown to either directly or indirectly affect the regulation of clock gene function. For example, the reduced forms of NAD^+^ and NADP^+^ (i.e., NADH and NADPH) increase the DNA-binding activity of the CLOCK:BMAL1 heterodimer in vitro [71]. Accordingly, inhibition of the pentose phosphate pathway, a major source of NADPH, prolongs the circadian period of clock gene expression in cultured cells [72]. Recently, it has also been reported that the circadian period of PER2 expression is lengthened by FAD through stabilization of CRY proteins [73]. Given that the levels of NAD^+^ and FAD oscillate in both tissues and cultured cells [73,74,75,76], the diurnal changes in these hydrogen carriers may play an important role in shaping the rhythmic expression of clock genes. The oscillation of hydrogen carriers seems to be under enzymatic control because both the mRNA and protein levels of genes encoding nicotinamide phosphoribosyltransferase (NAMPT) and riboflavin kinase (RFK), which are the rate-limiting enzymes of the NAD^+^ salvage pathway and FAD biosynthesis, respectively, vary dynamically in a circadian fashion in the murine liver [73,74,75]. Although the molecular mechanisms underlying the rhythmic expression of *Rfk* remain unclear, the transcription of *Nampt* has been reported to be directly regulated by the CLOCK:BMAL1 heterodimer through the E-box in its promoter [74,75]. Collectively, these findings reveal that cellular redox affects clock function which in turn participates in the circadian regulation of redox state, suggesting a mutual relationship between controls of cellular redox and clock function.

NAD^+^-consuming enzymes have also been shown to link energy metabolism with the molecular clock (Figure 2). Sirtuin 1 (SIRT1), whose activity relies on the NAD^+^ level, deacetylates histones and a wide variety of proteins, including those that regulate glucose and lipid metabolism [77,78,79,80,81]. SIRT1 forms a complex with CLOCK:BMAL1 and promotes the deacetylation of BMAL1, which then alters the transcriptional activity of CLOCK:BMAL1 [82,83]. As mentioned earlier, since DEC1 and DEC2 are transactivated by CLOCK:BMAL1, it is possible that SIRT1 indirectly regulates DEC1 and DEC2 levels through BMAL1 deacetylation. SIRT1 also deacetylates and degrades PER2 [83]. In addition, SIRT1 deacetylates and activates PGC-1α (PPARγ coactivator 1-alpha) [81,84], a transcriptional coactivator that promotes *Bmal1* expression through coactivation of ROR family members, including RORα and RORγ [85]. Consistent with these findings, deletion of the *Sirt1* gene leads to a significant reduction in the expression level of *Bmal1* [83,86]. Interestingly, mice with brain-specific knockout of *Sirt1* showed a lengthened circadian period of locomotor activity [86], which further indicates that SIRT1 is linked to clock function. In addition to SIRT1, poly (ADP-ribose) polymerase 1 (PARP-1), which exerts its physiological function by transferring ADP-ribose subunits from NAD^+^ to its target proteins, has also been reported to modulate the function of the molecular clock. PARP-1 binds and poly (ADP-ribosyl) ates the CLOCK protein, thereby reducing the DNA binding activity of CLOCK: BMAL1 [87]. Importantly, this phenomenon is observed to occur in a circadian fashion. It is possible that PARP-1 indirectly decreases DEC1 and DEC2 levels through the CLOCK:BMAL1 reduction.

Nocturnin which encodes circadian deadenylase is regulated by CLOCK:BMAL1, and crosstalk circadian and metabolism [88]. Nocturnin plays important roles in lipid metabolism, adipogenesis, and glucose homeostasis, involving circadian clock [88].

Taken together, these findings suggest that the clock can sense cellular redox through changes in the level of hydrogen carriers and/or the activity of NAD^+^-consuming enzymes.

### 6.2. Cellular Energy Status

**Figure 3:**
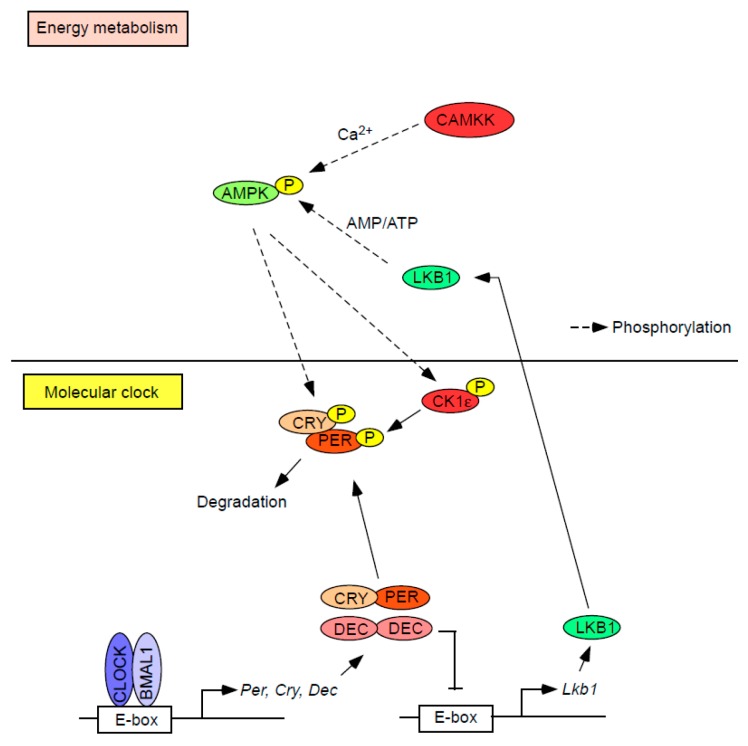
Interplay between the regulation of bioenergy and the molecular clock. Activated AMPK directly or indirectly phosphorylates core circadian repressors (i.e., PER/CRY). DEC, another circadian repressor, suppresses the transcription of *Lkb1* which codes for a serine-threonine kinase that directly phosphorylates and activates AMPK, thus forming a reciprocal relationship between regulators of bioenergy and the circadian clock.

In addition to cellular redox, changes in the intracellular level of energy resources (i.e., ATP) are sensed by the molecular clock. AMPK has been proposed as the key molecule that mediates the ATP-sensing property of the molecular clock [89,90]. AMPK is a heterotrimeric protein composed of a catalytic α and regulatory β and γ subunits, and each of these subunits is encoded by either 2 (α1 and α2 or β1 and β2) or 3 genes (γ1, γ2, and γ3) [91,92,93]. AMPK is activated when a threonine residue (Thr172) within the α subunit is phosphorylated by upstream kinases. These include liver kinase B1 (LKB1) and calmodulin-dependent protein kinase kinase (CaMKK). LKB1 is thought to be constitutively active and promotes phosphorylation of Thr172 when AMP binds to the AMPK-γ subunit, while CaMKK activates (i.e., phosphorylates) AMPK in response to a rise in intracellular Ca^2+^, independent of changes in the AMP/ATP ratio. Due to the energy-dependent phosphorylation of AMPK by LKB1, AMPK has been considered a cellular energy sensor. Importantly, it has been reported that activated AMPK directly or indirectly interacts with the molecular clock (Figure 3) [89,90]. For instance, AMPK directly phosphorylates CRY1 and indirectly phosphorylates PER2 by activating (i.e., phosphorylating) CKIε (casein kinase Iε) [94,95], which leads to the degradation of both CRY1 and PER2. Therefore, it could be speculated that a defect in the function of AMPK causes dysregulation of the circadian clock. Indeed, mice deficient in the *Ampkα1* gene show a shorter circadian period of locomotor activity [96]. Moreover, the rhythmic expression of clock genes in peripheral tissues is altered in mice lacking either *Ampkα1* or *Ampkα2* [96].

While the regulation of the molecular clock is modulated by AMPK, the molecular clock in turn regulates AMPK activity [43,97]. We have recently reported that DEC1 directly binds to the E-box box of the *Lkb1* promoter and suppresses the expression of the LKB1 protein, thereby reducing the phosphorylation of AMPK [97]. We also reported that the phosphorylation of AMPK is increased in the liver and lung in *Dec2* knockout mice, indicating that DEC2 may also suppress AMPK activity [43], although the precise mechanism remains to be elucidated. Of note, phosphorylation of AMPK is rhythmic and is inversely correlated with the rhythmic expression of DECs [20,96,97], further indicating that AMPK is a downstream target of DECs. Taken together, these findings suggest a mutual relationship between the function of the molecular clock and AMPK activity. It will be important to identify other molecules that link clock function and changes in the cellular energy balance.

### 6.3. Glucose Metabolism

In vitro studies have shown that the change in the glucose level also affects the function of the molecular clock. By using rat-1 fibroblasts, Hirota et al. demonstrated that glucose down-regulates the transcription of the *Per1* and *Per2* genes [98]. We and others have also reported that in cultured cells, *Dec1* expression is increased by glucose supplementation, while glucose depletion decreases both DEC1 and DEC2 protein levels [43,99]. These findings are consistent with in vivo studies that fasting decreases and re-feeding increases *Dec1* expression in the murine liver [68,69,70]. The concept that glucose is a key nutrient cue that alters clock gene function has been further supported by a series of animal studies. Among food macronutrients, glucose has been shown to alter the phase of the rhythm of locomotor activity in mice [100,101]. In addition, Oike et al. showed that intraperitoneal administration of glucose with amino acids increases the mRNA expression levels of *Per1*, *Per2*, *Dec1*, and *Dec2* in the liver in fasted mice [70]. While the precise mechanisms underlying the glucose-induced alteration in clock function remain unknown, transcriptional regulators have been proposed to mediate the effect of glucose on clock genes [98]. Liver X receptor (LXR), a glucose-activated transcription factor [102], may be an attractive candidate transmitter of the glucose signal to the molecular clock because LXR was found to induce *Dec1* expression by binding its promoter [69].

In addition to glucose, insulin was also found to affect the function of the molecular clock. Recent studies have shown that insulin promotes the phosphorylation of the BMAL1 protein by Akt, thereby suppressing its transcriptional activity [103]. Furthermore, several studies demonstrated that insulin increases the expression levels of *Per1*, *Per2*, *Dec1*, and *Dec2* in cultured cells [104,105,106,107]. While the molecular mechanisms underlying insulin-induced expression of these clock genes remain to be elucidated, insulin signalling pathways may play an important role in the regulation of the molecular clock because insulin-induced expression of *Dec1* and *Dec2* was found to be inhibited by the blockade of phosphoinositide 3-kinase, protein kinase C, or mammalian target of rapamycin [104,105,106].

We showed that high-fat diet induced the insulin and glucose levels [65]. As described above, insulin and glucose induced *Dec1* and *Dec2* expression, respectively. We speculate that high-fat diet induces *Dec1* and *Dec2* expression through insulin and glucose supplement.

### 6.4. Lipid Metabolism

The observation that the regulation of PPARs is under the control of clock proteins has led to the idea that the function of the molecular clock is linked to lipid metabolism [107]. Both DEC1 and DEC2 were found to regulate adipogenesis by repressing the transcription of a gene encoding PPARγ, the master transcriptional regulator of adipogenesis [63,108,109]. Accordingly, *Dec1* overexpression has been shown to suppress adipocyte differentiation [110]. DEC1 does not directly bind to the Pparγ promoter, but exerts its repressive effect on Pparγ expression by interacting with DNA-bound CCAAT/enhancer binding protein [63,108]. In addition to DEC proteins, other clock proteins have also been shown to regulate adipocyte differentiation [111]. PER2 inhibits the recruitment of PPARγ to its target gene promoters, and a defect in the *Per2* gene increases adipogenesis in cultured cells [112]. In contrast, knockdown of the *Bmal1* gene was found to suppress adipocyte differentiation in 3T3-L1 cells [55], although in vivo studies have shown contradictory results, specifically, that mice with adipocyte-specific knockout of the *Bmal1* gene develop obesity [113]. It should be noted that the function of PPARγ could in turn affect the molecular clock. The transcription of a gene coding for REV-ERBα, a circadian transcriptional repressor, was found to be induced by PPARγ through the direct repeat-2 response element in the *Rev-erbα* gene [114].

In addition to PPARγ, PPARα, the major regulator of fatty acid oxidation in the liver, has also been reported to interact with the molecular clock. The interconnection between PPARα and the circadian clock was first indicated in studies showing that the expression of PPARα is rhythmic at both the mRNA and protein levels [115]. Subsequently, PPARα was found to be a direct target of clock genes [116,117]. The CLOCK:BMAL1 heterodimer activates the transcription of the *Pparα* gene by binding to the E-box within the gene promoter [116]. PER2 also interacts with the PPARα protein through its LXXLL motifs [117]. Similar to the case of PPARγ, PPARα in turn regulates the transcription of clock genes. PPARα transactivates the *Bmal1* and *Rev-erbα* genes via PPAR response elements located in the promoters of these genes [118,119].

### 6.5. Hypoxia

Changes in tissue oxygen levels have also been shown to alter clock gene function. Both *Dec1* and *Dec2* expression were found to be induced by hypoxic conditions in cultured cells [120,121,122,123]. Promoter analyses have revealed that hypoxia-inducible factor-1α (HIF-1α), a master regulator of oxygen homeostasis, activates the transcription of the *Dec1* and *Dec2* genes by binding to the hypoxia-response elements (HREs) present within the promoters of these genes [121]. More recently, an additional interrelationship between hypoxia and clock gene function has emerged from studies by Peek et al. [124], who demonstrated that HIF-1α binds to the E-box within the promoters of *Per2* and *Cry1*. While HIF-1α regulates the transcription of core clock genes, it has also been shown that the clock proteins in turn interact with the HIF-1α protein. For instance, DEC2 binds to HIF-1α and decreases the binding of HIF-1α to HREs to activate the transcription of the HIF target genes [120]. Co-expression of HIF-1α and BMAL1 has also been shown to significantly transactivate HRE [124], further indicating the interaction between the clock proteins and HIF-1α. These findings are consistent with the observation that HIF-1α target genes show diurnal rhythms in their expression [120,125]. Because the alternation of HIF-1α and DEC expression is closely associated with tumor progression [120,126], it would be of interest to examine whether conditional *Hif-1α* and *Dec* knockout mice implanted tumor cells affect the circadian rhythm and metabolism. Collectively, these studies suggest that cellular oxygen homeostasis is tightly coupled to the regulation of daily rhythms in various physiological processes.

## 7. Conclusions

The clock genes *Dec1*, *Dec2*, and *Bmal1* are now recognized as important mediators that integrate the function of the circadian and metabolic systems. Dysfunction of the clock genes can therefore provoke both circadian and metabolic disorders. Further efforts to understand how *Dec1*, *Dec2*, and *Bmal1* sense energy balance and how energy imbalance impacts the function of these clock genes will provide insight into pathophysiologic interactions between circadian disorders such as sleep disturbances and metabolic disorders such as diabetes and obesity in humans.
